# Supplementary figures and images for: The defense island repertoire of the Escherichia coli pan-genome
Source: PLoS Genet. 2023 Apr 6;19(4):e1010694. doi: 10.1371/journal.pgen.1010694 (PMC10121019; doi:10.1371/journal.pgen.1010694)

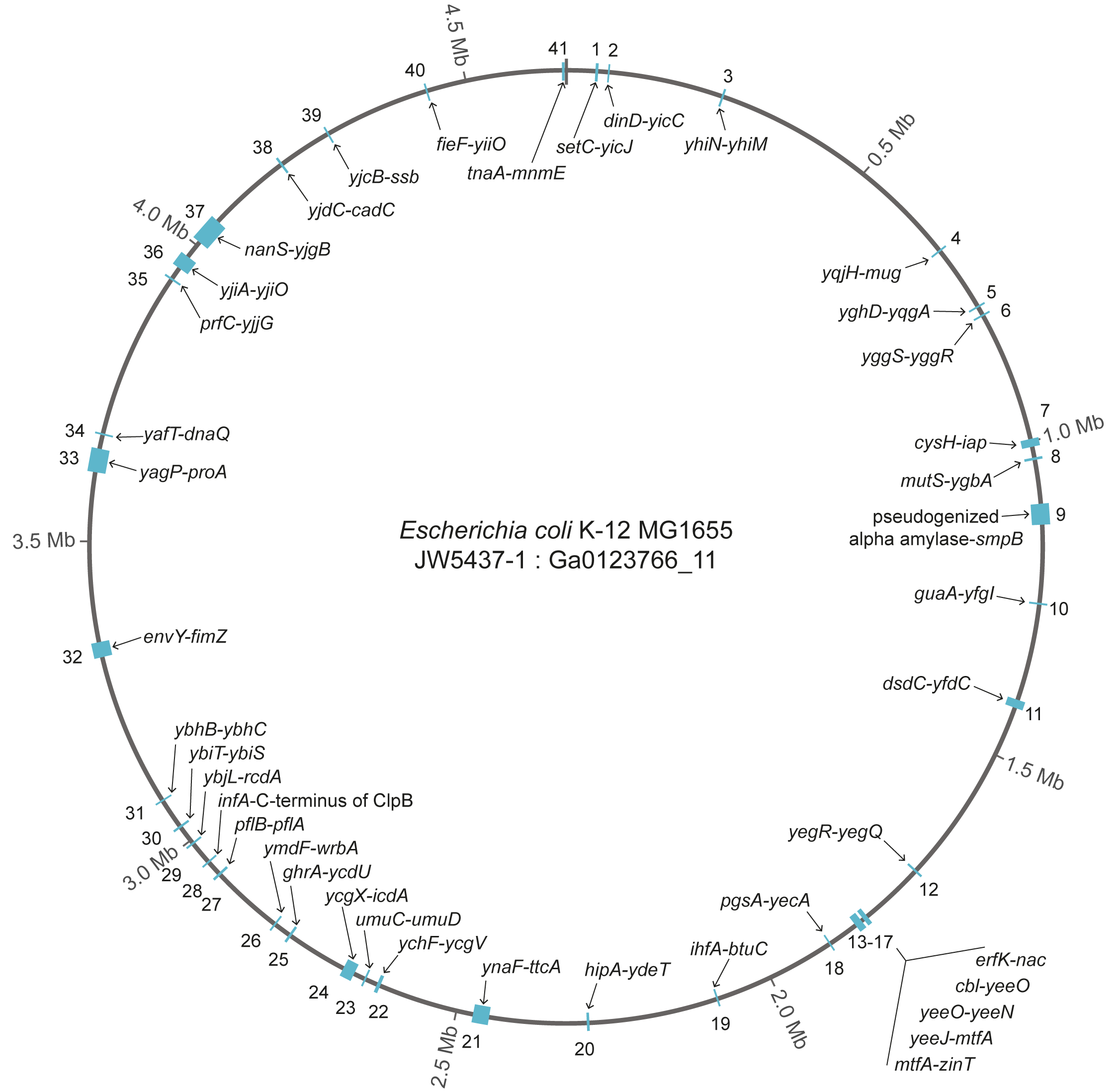

Supplement: S1 Fig — Numbers outside the ring indicate hotspot number. Flanking core genes for each hotspot are indicated on the inside. Blue ticks indicate the position of the hotspot in the E. coli K-12 reference genome, with thicker ticks reflecting hotspots that are occupied in K-12. When a given hotspot is occupied in the K-12 genome, tick thickness is proportional to the size of the island inserted at the hotspot. (TIF) [file pgen.1010694.s001.tif]

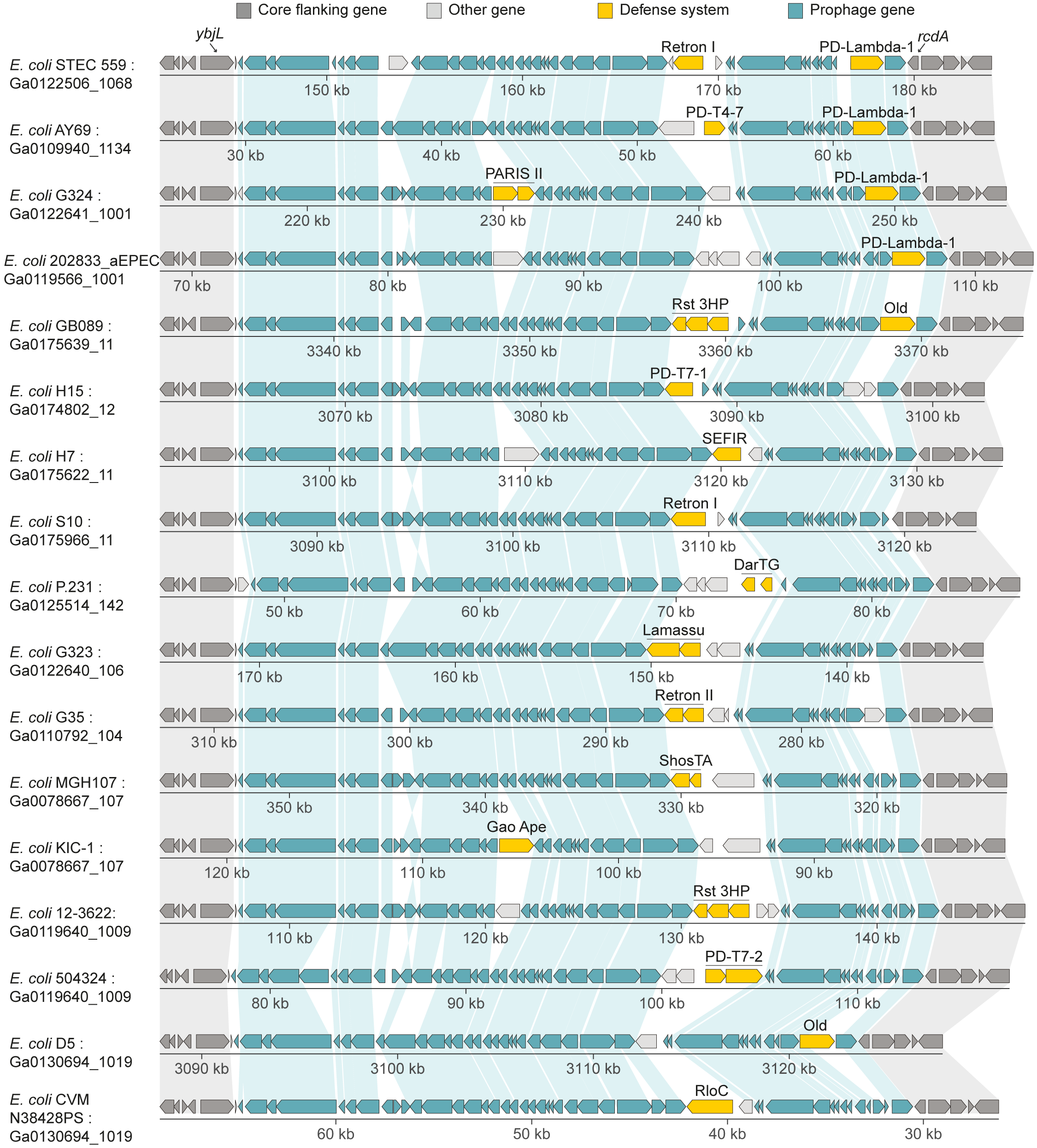

Supplement: S2 Fig — Shown are multiple examples of Felsduoviruses that integrate at hotspot #29. Defense systems are marked in yellow. Genome similarity was visualized using Clinker [1]. 1. Gilchrist CLM, Chooi YH. clinker & clustermap.js: automatic generation of gene cluster comparison figures. Bioinformatics. 2021;37: 2473–2475. (TIF) [file pgen.1010694.s002.tif]

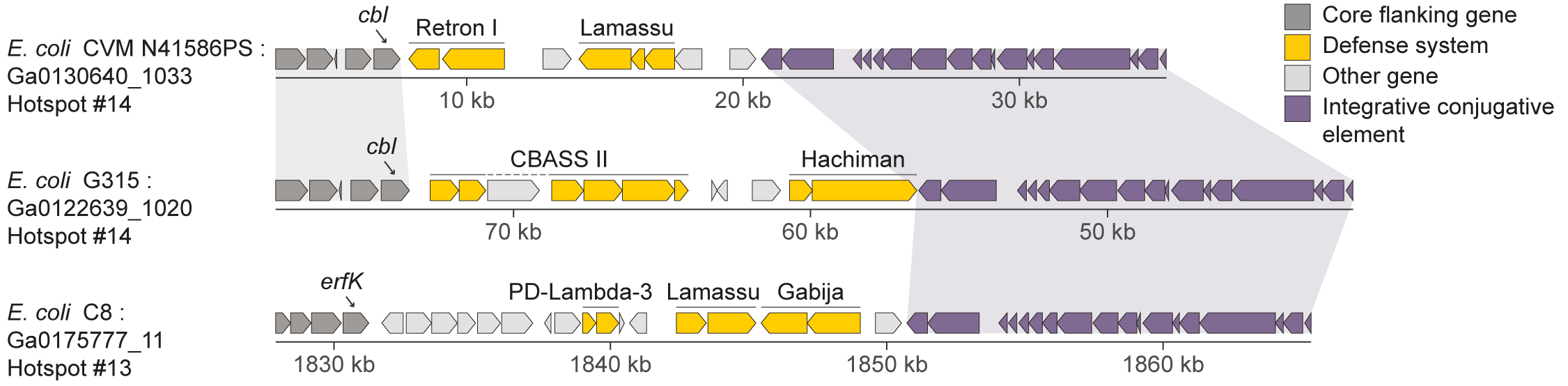

Supplement: S3 Fig — Instances of this element carry defense systems and are integrated at hotspots #13 and #14. Grey shading indicates conservation of core genes flanking the integration position at hotspot #14, purple shading indicates conservation of the integrative conjugative element (ICE) at these loci. Only part of the island is shown for space constraints. (TIF) [file pgen.1010694.s003.tif]
